# Supplementary material for: Maternity waiting homes utilization and associated factors among childbearing women in rural settings of Finfinnee special zone, central Ethiopia: A community based cross-sectional study
Source: PLoS One. 2022 Mar 17;17(3):e0265182. doi: 10.1371/journal.pone.0265182 (PMC8929623; doi:10.1371/journal.pone.0265182)
Supplement: S1 File — (DOCX) [file pone.0265182.s002.docx]

**English version and local language**

**Part I: Socio-demographic questionnaires**

| **S.N** | **Question** | **Coding categories** | **Remark** |
| --- | --- | --- | --- |
| **101** | Age in years | 1. _____________ |  |
| **102** | What is your Religion? | 1. Orthodox 2. Muslims  3. Protestant 4. Catholic  5. Others______ |  |
| **103** | What is your ethnicity? | 1. Oromo 2. Amhara 3. Gurage 4. Tigre 5. Others____________ |  |
| **104** | What is your marital status? | 1. Married 2. Single 3. Widowed 4. Divorced |  |
| **105** | What is your educational status? | 1. Unable to read and write 2. Only read and write 3. Primary education (grade 1-8) 4. Secondary education (grade 9 -10) 5. Preparatory education (grade 11-12) 6. Higher education (10+2, Diploma, Degree, Masters, PhD) |  |
| **106** | What is your husband educational status? | 1. Unable to read and write 2. Only read and write 3. Primary education (grade 1-8) 4. Secondary education (grade 9 -10) 5. Preparatory education (grade 11-12) 6. Higher education (10+2, Diploma, Degree, Masters, PhD ) |  |
| **107** | What is your occupation? | 1. Housewife 2. Farmer  3. Merchant 4. Students  5. Government employee  6. Others (Specify) __________ |  |
| **108** | What is your husband occupation? | 1. Farmer 2. Merchant 3. Students   4. Government employee  5. Others (Specify) __________ |  |
| **109** | Which of these materials exist in your house? | |  |
|  | Farmland | 1. Yes 2. No |  |
|  | Cow | 1. Yes 2. No |  |
|  | Ox | 1. Yes 2. No |  |
|  | Horse | 1. Yes 2. No |  |
|  | Donkey | 1. Yes 2. No |  |
|  | Goat | 1. Yes 2. No |  |
|  | Sheep | 1. Yes 2. No |  |
|  | Chicken | 1. Yes 2. No |  |
|  | Has electricity | 1. Yes 2. No |  |
|  | Solar light | 1. Yes 2. No |  |
|  | Kerosene lamp | 1. Yes 2. No |  |
|  | Has refrigerator | 1. Yes 2. No |  |
|  | Pipe Water | 1. Yes 2. No |  |
|  | Improved Latrine | 1. Yes 2. No |  |
|  | Has car | 1. Yes 2. No |  |
|  | Bajaj | 1. Yes 2. No |  |
|  | Has motorcycle | 1. Yes 2. No |  |
|  | Has television | 1. Yes 2. No |  |
|  | Has radio | 1. Yes 2. No |  |
|  | Mobile phone | 1. Yes 2. No |  |
|  | Chair | 1. Yes 2. No |  |
|  | Table | 1. Yes 2. No |  |
|  | Bed with Matters | 1. Yes 2. No |  |
|  | Health insurance | 1. Yes 2. No |  |
|  | Others (specify) ________ |  |  |
| **110** | The main material of the roof (record your observation) | |  |
|  | Hatch/leaf/ mud | 1. Yes 2. No |  |
|  | Has corrugate iron roofing | 1. Yes 2. No |  |
|  | Has roofing tiles | 1. Yes 2. No |  |
|  | Has other roofing | 1. Yes 2. No |  |
| **111** | The main material of the walls (record observation.) | |  |
|  | No walls | 1. Yes 2. No |  |
|  | Plywood | 1. Yes 2. No |  |
|  | Cement | 1. Yes 2. No |  |
|  | Rudimentary walls | 1. Yes 2. No |  |
|  | Reused wood | 1. Yes 2. No |  |
|  | Stone with cement | 1. Yes 2. No |  |
|  | Stone with mud | 1. Yes 2. No |  |
|  | Finished walls | 1. Yes 2. No |  |
|  | Cement block | 1. Yes 2. No |  |
|  | Covered adobe | 1. Yes 2. No |  |

**Part II: Individual-level factors towards MWH utilization**

**A. Obstetric History**

| **S.N** | **Question** | | | **Coding categories** | **Remark** |
| --- | --- | --- | --- | --- | --- |
| **201** | How many pregnancies do you have experiences? | | | 1. _____________ |  |
| **202** | The number of live births? | | | 1. _____________ |  |
| **203** | Did you receive ANC follow up including medical care during your recent pregnancy at the health facility? | | | 1. Yes 2. No | if No skip to no. 207 |
| **204** | If yes to question No. 203, how many times have you got ANC to follow up visit? | | | 1. 1^st^  2. 2^nd^   3. 3^rd^ 4. 4^th^ |  |
| **205** | If yes to question number 203, What health services did you receive when you visited the health facility during pregnancy? (multiple responses) | | | 1. Physical examination (including weight, blood pressure, heart rate) 2. Gynecological examination 3. Ultrasound 4. Blood tests including HIV/STD 5. Nutritional supplements 6. Tetanus vaccine 7. Others specify______________ |  |
| **206** | Did you have a birth preparedness plan for recent delivery? | | | 1. Yes 2. No |  |
| **207** | Delivered in a health facility? | | | 1. Yes 2. No | If No skip to no. 209 |
| **208** | If yes to question number 207, at which facility did you gave birth? | | | 1. Government Hospital 2. Government Health center 3. Health Post 4. Private clinic 5. Private Hospital 6. NGO health institute 7. Others specify_______________ |  |
| **209** | If no to question No 207 Where do you gave birth? | | | 1. Home 2. On the way to health facilities 3. Other, specify_______________ |  |
| **210** | Who assisted with the delivery? | | | 1. Doctor 2. Nurse/midwife   3. HEW  4. TBA  5. Untrained traditional birth attendant  6. Other, specify_____________ |  |
| **211** | Did you have PNC follow up for the recent delivery/birth? | | | 1. Yes   2. No | If no skip to No 213 |
| **212** | If yes to question No.211, Which PNC follow up visit did you got? | | | 1. The 1^st^ PNC at 24 hours after birth. 2. The 2^nd^ PNC b/n 49-72hrs. 3. The 3^rd^ PNC b/n 73hrs-7 days. 4. The 4^th^ PNC b/n 7^th^day & 6^th^ week. |  |
| **B** | **Information about Maternity Waiting Home** | | | |  |
| **S.N** | **Question** | | | **Coding categories** | **Remark** |
| **213** | Do you have information about MWH? | | | 1. Yes 2. No | if no skip to Q220 |
| **214** | If yes to question number 213, Where is the source of information? | | | 1. Other women  2. Husband  3. Nurse/ midwife  4. HEW  5. Others_______________ |  |
| **215** | Was the information given to you clear? | | | 1. Yes 2. No |  |
| **216** | What are the benefits of using MWH? (Multiple Response is possible) | | | 1. To get a healthy child 2. To get healthy Mother 3. To prevent morbidity and mortality of new born and mother 4. To get better health care from health professionals 5. Others specify________________ |  |
| **217** | Did anyone recommend you to use the MWH during one of your ANC visits? | | | 1. Yes 2. No | if no skip to Q 220 |
| **218** | If yes to question number 217, Who recommend you to use MWH? | | | 1. Other women 2. Husband 3. Family 4. Nurse/ midwife 5. HEW 6. Others_______________ |  |
| **219** | In what Gestational period do they recommend you to use MWH? | | | 1. Before 38 wks of Gestational age 2. After 38 wks of gestational age |  |
| **C.** | **Experience of MWH utilization** | | | |  |
| **S.N** | **Question** | | | **Coding categories** | **Remark** |
| **220** | Have you used MWH for your recent delivery/birth? | | | 1. Yes 2. No | If No skip to # 225 |
| **221** | If your answer to question no 220 is yes, what is your reason to use MWH? | | | 1. Fear of labour illness & complications 2. To get enough rest and free from workload 3. To get better health care from health professionals 4. To prevent death related to delivery 5. To get healthy child 6. Others specify________________ |  |
| **222** | How many days have you stayed in MWH? | | | 1. Less than 15 days 2. 15 days and More |  |
| **223** | Services available and received during stay (Multiple Response is possible) | | | 1. Bedding 2. Meals 3. Coffee 4. Latrines 5. Bathing facilities 6. Clean water 7. Electricity/lighting 8. Midwifery or Health professional check-up 9. Others specify________________ |  |
| **224** | Can you explain how it was for you to wait in MWH? | | | 1. Good 2. Not Good | Proceed to Q 301 |
| **225** | What are your reasons for not using an MWH? (For mothers who did not use MWH) (Multiple responses is possible) | | | 1. Absence of MWH 2. Lack of information and didn’t know the availability of MWH 3. No permission from husband/family 4. No money 5. Poor quality MWH 6. Absence of skilled attendant 7. Culturally not supported 8. Distance from home 9. Absence of transportation to and from the MWH 10. Not providing food while staying at MWH 11. Child care by others while staying at MWH is difficult 12. Attendant being away from work 13. No problem in previous Pregnancy 14. Others specify____________ | Proceed to Q. No 408 and finish it |
| **Part III. Health facility-related factors towards MWH utilization (for those who use MWH)** | | | | | |
| **S.N** | | **Question** | | **Coding categories** | **Remark** |
| **A** | | **Physical & economic accessibility** | | |  |
| **301** | | Perceived ease or difficulty of finding transport to reach a facility in case labour starts at home? | | 1. Easy 2. Difficult |  |
| **302** | | How long does it take you to travel to the nearest health facility? | | 1. Less than 30 minutes 2. 30 minutes to 1 hour 3. 1 hour to 1 ½ hour 4. 1 ½ to 2 hours 5. More than 2 hours |  |
| **303** | | Which Mode of transport do you use to go to the nearest Health facility in case of emergency? | | 1. Walking/carried 2. Horse 3. Vehicles |  |
| **304** | | Transport to and from the MWH? | | 1. Affordable 2. Not affordable |  |
| **305** | | How did you organize the money (for the transport? MWH) | | 1. Husband/partner 2. Family member 3. Respondent 4. Others (Specify)_______________ |  |
| **306** | | What was the average amount of time that you waited to see medical staff when you visited the health facility? | | 1. Less than 30 minutes 2. 30 minutes to 1 hour 3. 1 hour to 1 ½ hour 4. 1 ½ to 2 hours 5. More than 2 hours |  |
| **B.** | | **Client satisfaction** | | |  |
| **307** | | How satisfied were you with the care you received from MWH? | 1. Satisfied 2. Not Satisfied | |  |
| **308** | | What were the reasons for your dissatisfaction? (open-ended) | | _____________________________ |  |
| **309** | | Waiting time to get MWH service | | 1. Up to 30 minutes 2. More than 30 minutes |  |
| **C** | | **Problems with MWH:** | | |  |
| **310** | | Availability of cooking area | | 1. Yes 2. No |  |
| **311** | | Access to a cooking area | | 1. Yes 2. No |  |
| **312** | | Boredom | | 1. Yes 2. No |  |
| **313** | | Cleanliness | | 1. Good 2. Bad |  |
| **314** | | Crowdedness | | 1. Yes 2. No |  |
| **315** | | Cultural Appropriateness | | 1. Good 2. Bad |  |
| **316** | | Management Oversight | | 1. Good 2. Bad |  |
| **317** | | Quality and Safety | | 1. Good 2. Bad |  |
| **318** | | Presence of Staff 24 hrs | | 1. Yes 2. No |  |
| **319** | | Friendliness of Staff | | 1. Yes 2. No |  |
| **D** | | **Characteristics of MWH** | |  |  |
| **320** | | Separate bed available | | 1. Yes 2. No |  |
| **321** | | Availability of curtains for privacy | | 1. Yes 2. No |  |
| **322** | | Availability of medical equipment and medicine. | | 1. Yes 2. No |  |
| **323** | | Sleep under Mosquito Net | | 1. Yes 2. No |  |
| **324** | | Oriented to Rules 24 hrs | | 1. Yes 2. No |  |
| **325** | | The family are welcome to stay with women at MWH | | 1. Yes 2. No |  |
| **326** | | Extra space for the family to stay with you at MWH | | 1. Yes 2. No |  |
| **327** | | Access to food | | 1. Yes 2. No |  |
| **328** | | Access to water | | 1. Yes 2. No |  |
| **329** | | Access to Light | | 1. Yes 2. No |  |
| **330** | | Bathing Area | | 1. Yes 2. No |  |
| **331** | | Safe Space for Belongings | | 1. Yes 2. No |  |
| **332** | | Health Education sessions | | 1. Yes 2. No |  |
| **333** | | Availability of TV/Radio | | 1. Yes 2. No |  |

**Part IV. Household and Community-related factors towards MWH utilization**

| **S. N** | **Question** | **Coding categories** | **Remark** |
| --- | --- | --- | --- |
| **401** | Have you discussed MWH with your husband or family? | 1. Yes 2. No |  |
| **402** | Have you planned or decides to use MWH during the recent pregnancy? | 1. Yes 2. No | If no skip to 404 |
| **403** | By who was the decision made to come to the MWH? | 1. Husband 2. Woman (myself) 3. Family 4. TBA 5. HEW 6. Nurses/MW |  |
| **404** | Does your husband support your idea to use MWH? | 1. Yes 2. No |  |
| **405** | Did you have to make arrangements at home or with family before you went there? | 1. Yes 2. No |  |
| **406** | Attendant during MWH stay? | 1. Husband 2. Mother/Mother-in-law 3. Other family member _________ |  |
| **407** | Attendant being away from work during MWH stay? | 1. Not possible 2. Possible |  |
| **408** | Social support including a child and household care at home during MWH stay came from? | 1. Husband/partner 2. Family member 3. Neighbours 4. Servant 5. No one |  |
| **409** | How do you see the overall importance of MWH? | 1. Important 2. Not important |  |

**THANK YOU FOR YOUR PARTICIPATION!**
