# Supplementary material for: Maternity waiting homes utilization and associated factors among childbearing women in rural settings of Finfinnee special zone, central Ethiopia: A community based cross-sectional study
Source: PLoS One. 2022 Mar 17;17(3):e0265182. doi: 10.1371/journal.pone.0265182 (PMC8929623; doi:10.1371/journal.pone.0265182)
Supplement: Attachment — Submitted filename: Response to Reviewers.docx [file pone.0265182.s004.docx]

**Point by point responses to reviewer’s comments**

**Dear Reviewer,**

Thank you very much for your comments and suggestions that help us to improve the quality of our manuscript. We tried to address your comments, suggestions and questions as follows.

1. The English used in the manuscript is not appropriate for PLOS ONE.

**Response: Dear reviewer,**

We tried to correct the English language write up and the grammatical errors of the whole manuscript with a trained English language expert.

2. It is not clear what the authors are communicating here. Are MWHs meant to improve access or they are strategies for improved access? What do the authors mean by “it is vital to use MWH for Ethiopia”? Improved access to service delivery alone does not solve the problem of high maternal mortality

**Response: Dear reviewer,**

Thank you for your concern. In Ethiopia, where maternal mortality is more prevalent and the most important cause is the inaccessibility of health services, strategies like MWH will be crucial. Moreover, we have corrected the sentence. Please see page 2 lines 24-25.

3. Line 51: Could authors write world health organization appropriately?

**Response: Dear reviewer,**

We corrected it as per the suggestion. Please see page 4 line 52.

4. Line 54-56: Sentence is confusing and needs to be revised

**Response: Dear reviewer,**

We corrected it as per the suggestion. Please see page 4 lines 55-58.

5. Line 60: A bracket is opened but was not closed

**Response: Dear reviewer,**

We corrected it as per the suggestion. Please see page 4 line 60.

6. Line 70-71: Sentence does not make any sense

**Response: Dear reviewer,**

We corrected it as per the suggestion. Please see page 5 lines 71-75.

7. Line 71-75: Authors can highlight the determinants of intention to use MWHs without having to refer to the variable names as they appeared in the study (ies). For example, attended ANC can be replaced with something like women who attended ANC or use of ANC. Authors should revise to align with the sentence structure used.

**Response: Dear reviewer,**

We corrected it as per the suggestion. Please see page 5 lines 71-75.

8. Line 79: Where are the qualitative studies? I believe references 14-16 are about utilization and not intention to use.

**Response: Dear reviewer,**

We corrected it as per the suggestion. Please see page 5 line 82.

9. Line 93-94: Authors should break the sentence and continue with a new one.
    **Response: Dear reviewer,**

We corrected it as per the suggestion. Please see page 6 lines 93-94.

10. Does it mean the 9 health centers do not have MWHs?

**Response: Dear reviewer,**

Yes, the rest (9) of the health centres have no MWHs.

11. Line 107: delete far. Whether far or near is determined by the distance from the reference point

**Response: Dear reviewer,**

We corrected it as per the suggestion. Please see page 6 line 107.

12. Line 112-114: in the introduction, a utilization rate of 7% was quoted, but a 38.7% is quoted for the sample size? Why did authors choose the latter instead of the former to calculate the sample size?

**Response: Dear reviewer,**

Using the single proportion formula we calculated the final sample size separately and we got 158 employing 7% proportion of MWHs use which is calculated from only three districts of Jimma and we got 574 using 38.7% proportion of MWHs intended use. To get a more precise estimate, we used the latter one which gives the largest sample size. However, we also calculated the sample size for independent variables using the double population proportion formula and got 636 sample sizes. Hence, we used the largest sample size (636) as the final sample size of this study.

13. What is the single population formula and what is the purpose of the design effect?

**Response: Dear reviewer,**

A single population proportion formula is a formula used to calculate the sample size needed to estimate the proportion or percentage of an outcome of interest in a population from data of which the outcome consists of two categories (dichotomous). Based on it, we employed here the single proportion formula (**n = Z_α/2_^2^ *p*(1-p) / d^2^**) to estimate the sample size used to determine the proportion MWH utilization.

A design effect is a strategy used to adjust the sampling effect by increasing the sample size when employing other than simple random sampling (cluster sampling, systematic sampling, stratified sampling, multistage sampling). Hence, we employed here multistage sampling followed by systematic sampling. Therefore, to adjust for the sampling deviation from simple random we used a design effect of 1.5.

14. What justified the calculation of two sample sizes and ultimately settling on one? What do authors mean by sample size for factors?

**Response: Dear reviewer,**

The phrase “sample size for factors” was to mean that a sample size needs to be taken to measure the association of a single independent variable with the outcome variable. Therefore, calculating the sample size both for the outcome variable and the independent variables help to reach the largest sample size which is considered to include the other small sample sizes. Moreover, we corrected the phrase. Please take a look at page 7 line 116.

15. In the selection of health facilities, did the authors take into consideration health facilities that did not have MWHs? Obviously, that has a potential to influence utilization rate

**Response: Dear reviewer,**

Thank you for your very important point. We didn’t consider that because the absence of MWHs in the nearest health facility can be one of the reasons for not utilizing the MWHs. As we indicated in table 2, nine women responded that the absence of MWHs is one of the reasons not to use MWHs among women who didn’t utilize MWHs for their most recent birth.

16. There is evidence that the utilization of MWHs may not be the only solution to promoting maternal health in Ethiopia. From the study, even women who used MWHs did not get the full benefits; some did not get meals and some did not get clean water. What would be the benefit of using MWH to a woman when she is not assured of receiving what she is expected to get there?

**Response: Dear reviewer,**

Thank you. We believe that even if the services provided by the MWH is not satisfactory enough as intended by its objective, there are promising indicators that promote the utilization of MWH. Like for example in this study, 75.4% of women get health professionals' check-ups in the MWH. Therefore, even though MWHs utilisation, is not the only strategy for promoting maternal health, it will be highly important in reducing maternal mortality in risky and rural women with the difficulty of access to transportation.
