# Supplementary material for: Maternity waiting homes utilization and associated factors among childbearing women in rural settings of Finfinnee special zone, central Ethiopia: A community based cross-sectional study
Source: PLoS One. 2022 Mar 17;17(3):e0265182. doi: 10.1371/journal.pone.0265182 (PMC8929623; doi:10.1371/journal.pone.0265182)
Supplement: Attachment — Submitted filename: Response to Reviewer.docx [file pone.0265182.s005.docx]

**Point by point responses to reviewer’s comments**

**Dear Reviewer,**

We thank you for your comments, suggestions and questions that would be helpful in improving the quality of our manuscript. We tried to address all of your concerns as follows.

1. L55-L57 Edit the language.

**Response: Dear reviewer,**

We have edited the language construction. Please see page 4 line 53-55.

2. L57 - What does final week of pregnancy mean in this study as a term pregnancy is widely accepted as 37 completed weeks?

**Response: Dear reviewer,**

We have corrected it as per the suggestion. Please see page 4 line 54-55.

3. L60 - Remove the additional bracket.

**Response: Dear reviewer,**

We have corrected it as per the suggestion. Please see page 4 line 57-59.

4. L61 - What is the ideal maternal mortality? Place Ethiopia in the international perspective.

**Response: Dear reviewer,**

We have corrected the sentence. Please see page 4 line 57-59.

5. L61-L63 - What was the maternal mortality prior to implementation of the MWHs?

**Response: Dear reviewer,**

We have included the maternal mortality prior to implementation of the MWHs in the revised manuscript. Please see page 4 line 61.

6. L71-L80 - Many studies have been conducted on MWHs and the facilitators and barriers to use of the MWHs are known. The significance of the research problem is stated in L81-L88, but is not clear.

**Response: Dear reviewer,**

We have made clear the significance of the study. Please see page 4 and 5 from line 81- 91.

7. L92 - What is a community-based cross-sectional study? The study design is not described and applied to this study.

**Response: Dear reviewer,**

A community-based cross-sectional study design is one of the study designs, which can be descriptive or analytical that involves collecting data from a population from the community at one specific point in time. In this study, we have collected data from 15th October to 20th November 2019 among reproductive women who gave birth in the past six months of delivery before the actual data collection period in the rural settings of the Finfinnee special zone. To make more clarity, we have corrected the sentence. Please see page 5 line 94.

8. L93-L94 - The population was 649,403. Indicate the specific year.

**Response: Dear reviewer,**

We have indicated the year as per the suggestion. Please see page 5 line 95.

9. L97 - Approximately how many births are conducted in each kebele and what type of healthcare is present?

**Response: Dear reviewer,**

We have addressed you question. Please see page 5 line 99.

10. L107 - Why should the mothers live 9.5km away?

**Response: Dear reviewer,**

As we have indicated in the background, most Ethiopians live in rural settings where people are living far from health facility and we have used the cut-off point as referenced in another related study to operationalize how far is the pregnant women resides from the health facility. It has to be noted that MWHs service is mainly targeted to remote-residing pregnant women to break the geographical inaccessibility of obstetric care to reduce pregnancy complications and minimize perinatal mortality.

11. L119 - What is AOR 2.4?

**Response: Dear reviewer,**

It is to mean adjusted odds ratio. We have corrected it as per the suggestion. Please see page 7 line 126.

12. L128 - Correct the language in the sentence 'The sampling interval...'

**Response: Dear reviewer,**

We have edited the language construction of the sentence. Please see page 8 line 136-137.

13. L140 - There is a word missing in the sentence 'The obstetrical...'

**Response: Dear reviewer,**

We have corrected it as per the suggestion. Please see page 8 line 148.

14. L140 - L145 - How were the factors selected in the questionnaire? Describing the construction of the research tool is very important as it affect the validity and reliability of the study.

**Response: Dear reviewer,**

Thank you very much for your important point. However, in the “Data collection procedures and quality control” section we have detailed the description of the questionnaire development process and the data collection procedure in addition to the data quality assurance.

15. The results are well presented and clear.

**Response: Dear reviewer,**

Thank you very much for your appreciation.

16. L196-L202 - There is an overuse of adverts. Please remove some.

**Response: Dear reviewer,**

We have corrected it as per the suggestion. Please see page 13 line 206-209.

17. The role of the healthcare provider is still not clear as well as the benefits of use of the MWH for the women.

**Response: Dear reviewer,**

As we have indicated in the background section in detail, the benefit of the MWH is to facilitate easy access of pregnant women to obstetric care through making them near to the health facility. The service mainly targets risky pregnant women and pregnant women who live at very remote distances from health facilities. In this regard, the health facilities are expected to construct the MWHs and the health professionals should check them on a regular basis.

18. Perhaps create three headings in the discussion section

**Response: Dear reviewer,**

Thank you very much for your concern about the discussion and for the suggestions on the discussion sections. We have addressed the issues you raised in the discussion section, but we haven’t created the heading under the discussion section. However, each paragraph was written assuming to contain a specific idea. For example, the first paragraph is an introduction to the discussion section. The second paragraph is about the comparison of this study’s magnitude of MWH with the other local studies. The third paragraph is about comparison with other countries findings. The fourth paragraph is about services received by pregnant women. The fifth, sixth and seventh paragraph are about the factors associated with MWHs utilization. The last paragraph stated the limitations of the study. This form of discussion section writing is common in other studies including studies which were published in PLOS ONE journal.

19. L224-L225 - Correct the language in the sentence starting with 'The study showed...'

**Response: Dear reviewer,**

We have edited the language construction of the sentence. Please see page 16 line 231.

20. L225-L226 - The actual use in this study is compared to the intended use in other studies. There must be similar data available.

**Response: Dear reviewer,**

Thank you very much for your concern. However, we have used the available studies in Ethiopia, which were conducted on actual users of MWH, for comparison. But due to the limited number of studies on actual users, we have also compared our findings with intended users of MWH in other settings. Even though it is not the right way of comparison, we believe that there might not be huge difference among the studies settings with our study setting since all them were conducted in Ethiopia. In addition, comparison of the actual use of MWH with the intended use of MWH might also generate another evidence which will be used for policy influence.

21. L227-L228 - Correct the language in the sentence starting with 'The studies in Benchi Maji...'

**Response: Dear reviewer,**

We have corrected it as per the suggestion. Please see page 16 line 237-240.

22. L232-L234 - Correct the language

**Response: Dear reviewer,**

We have corrected it as per the suggestion. Please see page 16 line 233

23. L234-L235 - Correct the language. The sentence does not flow well.

**Response: Dear reviewer,**

We have corrected it as per the suggestion. Please see page 16 line 233-235.

24. L258 - Correct the language.

**Response: Dear reviewer,**

We have corrected it as per the suggestion. Please see page 17 line 268.

25. L256-L266 Correct the language of the sentence 'This might be the effect...'

**Response: Dear reviewer,**

We have corrected it as per the suggestion. Please see page 18 line 272-274.

26. L282 - Add 's' to the word 'result'.

**Response: Dear reviewer,**

We have added it as per the suggestion. Please see page 18 line 291.

27. L293 - 'Housewife women' is not appropriate English.

**Response: Dear reviewer,**

We have corrected it as per the suggestion. Please see page 19 line 301.

28. L302 - The word 'admire' is not appropriate English.

**Response: Dear reviewer,**

We have corrected it as per the suggestion. Please see page 19 line 310.
